# Hypolipidemic and insulin sensitizing effects of salsalate beyond suppressing inflammation in a prediabetic rat model

**DOI:** 10.3389/fphar.2023.1117683

**Published:** 2023-04-03

**Authors:** Martina Hüttl, Irena Markova, Denisa Miklánková, Iveta Zapletalova, Petr Kujal, Jan Šilhavý, Michal Pravenec, Hana Malinska

**Affiliations:** ^1^ Center for Experimental Medicine, Institute for Clinical and Experimental Medicine, Prague, Czech; ^2^ Department of Pharmacology, Faculty of Medicine and Dentistry, Palacky University, Olomouc, Czech; ^3^ Department of Pathology, Third Faculty of Medicine, Charles University, Prague, Czech; ^4^ Institute of Physiology, Czech Academy of Sciences, Prague, Czech

**Keywords:** salsalate, low-grade inflammation, prediabetes, lipid metabolism, cytochrome P450, oxidative stress

## Abstract

**Background and aims:** Low-grade chronic inflammation plays an important role in the pathogenesis of metabolic syndrome, type 2 diabetes and their complications. In this study, we investigated the effects of salsalate, a non-steroidal anti-inflammatory drug, on metabolic disturbances in an animal model of prediabetes—a strain of non-obese hereditary hypertriglyceridemic (HHTg) rats.

**Materials and Methods:** Adult male HHTg and Wistar control rats were fed a standard diet without or with salsalate delivering a daily dose of 200 mg/kg of body weight for 6 weeks. Tissue sensitivity to insulin action was measured *ex vivo* according to basal and insulin-stimulated ^14^C-U-glucose incorporation into muscle glycogen or adipose tissue lipids. The concentration of methylglyoxal and glutathione was determined using the HPLC-method. Gene expression was measured by quantitative RT-PCR.

**Results:** Salsalate treatment of HHTg rats when compared to their untreated controls was associated with significant amelioration of inflammation, dyslipidemia and insulin resistance. Specificaly, salsalate treatment was associated with reduced inflammation, oxidative and dicarbonyl stress when inflammatory markers, lipoperoxidation products and methylglyoxal levels were significantly decreased in serum and tissues. In addition, salsalate ameliorated glycaemia and reduced serum lipid concentrations. Insulin sensitivity in visceral adipose tissue and skeletal muscle was significantly increased after salsalate administration. Further, salsalate markedly reduced hepatic lipid accumulation (triglycerides −29% and cholesterol −14%). Hypolipidemic effects of salsalate were associated with differential expression of genes coding for enzymes and transcription factors involved in lipid synthesis (*Fas, Hmgcr*), oxidation (*Pparα*) and transport (*Ldlr*, *Abc* transporters), as well as changes in gene expression of cytochrome P450 proteins, in particular decreased *Cyp7a* and increased *Cyp4a* isoforms.

**Conclusion:** These results demonstrate important anti-inflammatory and anti-oxidative effects of salsalate that were associated with reduced dyslipidemia and insulin resistance in HHTg rats. Hypolipidemic effects of salsalate were associated with differential expression of genes regulating lipid metabolism in the liver. These results suggest potential beneficial use of salsalate in prediabetic patients with NAFLD symptoms.

## 1 Introduction

Prediabetic states characterized by insulin resistance, impaired glucose tolerance and disturbances in lipid metabolism, are accompanied by low-chronic inflammation that can contribute to the onset of T2DM and its complications. Thus, the amelioration of inflammation can have preventive and beneficial effects and represents a potential target for treatment of patients with metabolic syndrome, prediabetes or T2DM (Wu and Ballantyne, 2020).

Salicylate is one of the oldest drugs in clinical practice for treating inflammation. Salicylates exist in 2 forms: acetylated form - aspirin and non-acetylated form—salsalate. Unlike aspirin, which can lead to impaired coagulation and gastric irritation, especially at increased doses, salsalate is insoluble in the acidic gastric environment, is absorbed only in the small intestine and thus omits the gastric mucosa (Scheiman and Elta, 1990; Vane, 2002). Therefore, it is relatively safe for long-term clinical use.

In general, salicylates provide anti-inflammatory effect through the inhibition of cyclooxygenase (COX) enzymes. However, salsalate only mildly inhibits the COX pathway, but strongly inhibits NF-κB cascade and thus decreases the production of pro-inflammatory cytokines (Higgs, et al., 1987). It has been previously reported that anti-inflammatory action of salsalate can participate in improving glucose homeostasis (Liang, et al., 2015) suggesting that salsalate might have antidiabetic efficacy with minimal side effects.

In addition to its anti-inflammatory and anti-diabetic effects, salsalate can have other beneficial effects, in particular the effects on insulin sensitivity and lipid metabolism, some of which may be related to the anti-inflammatory effects of salsalate, others may be independent of these effects. Several recent studies confirmed the hypolipidemic effects of salsalate in diabetic mice (Nie, et al., 2017) and rats with inflammation induced by transgenic expression of human CRP (Trnovska, et al., 2017), as well as in individuals with abnormal glucose tolerance (Goldfine, et al., 2013) and patients with T2DM (Barzilay, et al., 2014). On the other hand, mechanisms responsible for metabolic effects of salsalate have not been fully elucidated. Accordingly, in this study, we investigated the effects of salsalate treatment on glucose and lipid metabolism, liver steatosis, glutathione system, inflammatory parameters and dicarbonyl stress in a rat prediabetic model with insulin resistance - a strain of hereditary hypertriglyceridemic (HHTg) rats, which exhibits most of symptoms of metabolic syndrome but without fasting hyperglycemia and obesity (Zicha et al., 2006). In contrast to the high-fat or high-sucrose diet induced obesity and subsequent symptoms, the strain of HHTg rats represents a model of insulin resistance with genetically determined presence of aggravated lipid spectrum in circulation as well as increased ectopic lipid storage and all in absence excessive obesity. Thus, this animal model could refer to non-obese patients suffer from metabolic syndrome symptoms as insulin resistance and severe dyslipidemia, especially. Given the low cost of salicylates, administration of salsalate to people with prediabetes and metabolic syndrome could be an additional and alternative therapy.

## 2 Methods

### 2.1 Animals and experimental procedure

All experiments were performed in accordance with the Animal Protection Law of the Czech Republic (311/1997), which is in compliance with the European Community Council recommendations for the use of laboratory animals (86/609/ECC) and were approved by the Ethical Committee of the Ministry of Education of the Czech Republic as well as by the Ethical Committee of the Institute for Clinical and Experimental Medicine. The study was carried out on 6-month-old male hereditary hypertriglyceridemic rats (HHTg) from the Institute for the Clinical and Experimental Medicine, Prague, Czech Republic as the non-obese prediabetic model and on Wistar rats (obtained from Charles River, Germany) as the control group. Rats were maintained in a 12-h light/12-h dark cycle room with the temperature at 22°C–25°C, allowed free access to food and water. Animals were randomized into groups (each group *n* = 8) and were fed standard laboratory diet (maintenance diet for rats and mice; Altromin, Lage, Germany) with or without salsalate (Cayman chemical, United States, Item 11911) supplementation at daily dose 200 mg/kg of body weight for 6 weeks. At the end of the experiments, rats were sacrificed after light anesthesia (zoletil 5 mg/kg b. wt.) in a postprandial state, blood and tissues were collected and stored at −80°C.

### 2.2 Analytical methods and biochemical analysis

Serum glucose, triglycerides and total cholesterol were determined using kits from Erba Lachema, (Czech Republic). HDL-cholesterol, non-esterified fatty acid (NEFA) with kits from Roche Diagnostic, (Germany). Serum insulin, glucagon, high molecular weight (HMV) adiponectin, hs CRP, leptin, MCP-1, IL-6 and TNFα concentrations were determined using rat ELISA kit (Mercodia, Sweden; MyBioSource, United States; eBioscience, United States; BioVendor, Czech Republic). Alanine aminotranspherase (ALT) and aspartate aminotraspherase (AST) enzyme activity was determined spectrophotometrically using diagnostic kit (Roche Diagnostics, Mannheim, Germany).

For the oral glucose tolerance test (oGTT), blood glucose was determined after a glucose load (3 g of glucose/kg b. wt.) administrated intragastrically after overnight fasting. Preceding the glucose load, blood was drawn from the tail at 0 min and then 30, 60 and 120 min thereafter.

To determine triglycerides and cholesterol content in the liver and skeletal muscle, samples were extracted using a chloroform/methanol mixture. The resulting pellet was dissolved in isopropyl alcohol, with triglycerides content determined by enzymatic assay (Erba-Lachema, Brno, Czech Republic).

### 2.3 Basal and insulin-stimulated glucose utilization in adipose tissue and skeletal muscle

For measurement of insulin-stimulated incorporation of glucose into lipids or glycogen, epididymal adipose tissue or skeletal muscle was incubated for 2 h in 95% O_2_ + 5% CO_2_ in Krebs-Ringer bicarbonate buffer, pH 7.4, containing 0.1 μCi/mL of ^14^C-U glucose, 5 mmol/L of unlabeled glucose, and 2.5 mg/mL of bovine serum albumin (Fraction V, Sigma, Czech Republic), with or without 250 μU/mL insulin. Extraction of lipids or glycogen was followed by a determination of insulin-stimulated incorporation of glucose into lipids or glycogen (Malinska, et al., 2015). In epididymal adipose tissue, basal and adrenaline-stimulated lipolysis were measured *ex vivo* based on the release of NEFA into the incubating medium.

### 2.4 Parameters of oxidative and dicarbonyl stress

Concentrations of reduced (GSH) and oxidized (GSSG) form of glutathione were determined using HPLC diagnostic kit with fluorescence detection (ChromSystems, Germany). Methylglyoxal concentrations were measured using the same HPLC method with fluorescence detection after derivatization with 1,2-diaminobenzene (Thornalley, et al., 1999). Activities of superoxide dismutase (SOD), glutathione peroxidase (GPx), glutathione reductase (GR), and glutathione transferase were analyzed using Cayman Chemicals assay kits (MI, United States). Concentration of conjugated dienes was determined by extraction in media (heptan: isopropanol = 2:1) and measured spectrophotometrically in heptan layer. Lipoperoxidation products were assessed based on levels of thiobarbituric acid-reactive substances (TBARS) by assaying the reaction with thiobarbituric acid.

### 2.5 Relative mRNA expression

Total RNA was isolated from the liver or adipose tissue using RNA Blue (Top-bio, Czech Republic). Reverse transcription and quantitative real-time PCR analyses were performed using the TaqMan RNA-to CT 1-Step Kit and TaqMan Gene Expression Assay (Applied Biosystems, United States) and carried out using a ViiA™ 7 Real Time PCR System (Applied Biosystems, United States). Relative expressions were determined after normalization against *β-actin* and *Hprt1* as an internal reference and calculated using the 2^−ΔΔCt^ method. The results were run in triplicates.

### 2.6 Histological evaluation

Liver tissue slices in middle sagittal plane and stomach specimens dissected along the greater curvature were fixed in 4% formaldehyde for 24 h, dehydrated and embedded in paraffin. The sections stained with hematoxylin eosin and picrosirius red were assessed in blind-test fashion. The non-alcoholic fatty liver disease (NAFLD) activity scores (NASs) as defined by Kleiner et al. (Kleiner, et al., 2005) were used to evaluate the liver parenchyma damage. Stomach samples were examined for any regressive or inflammatory changes.

### 2.7 Statistical analyses

Our hypothesis was to determine whether the effects of salsalate treatment would be different in prediabetic HHTg rats than in control Wistars. In this case, we used two-way ANOVA to test for strain effects, treatment effects and treatment vs. strain interaction, that determine whether the salsalate administration have different effect in prediabetic strain than in control Wistar strain. We used Fisher’s *post hoc* LSD test for multiple comparison to determine whether the effects of salsalate treatment are significant in prediabetic HHTg and in control Wistar strain. All data are expressed as mean ± SEM and were evaluated on StatSoft^®^ Statistica software (version 14, Statsoft CZ, Czech Republic). Normality distribution was tested by the Shapiro-Wilk test. Statistical significance was defined as *p* < 0.05.

## 3 Results

### 3.1 The effects of salsalate on body composition

**FIGURE 1:**
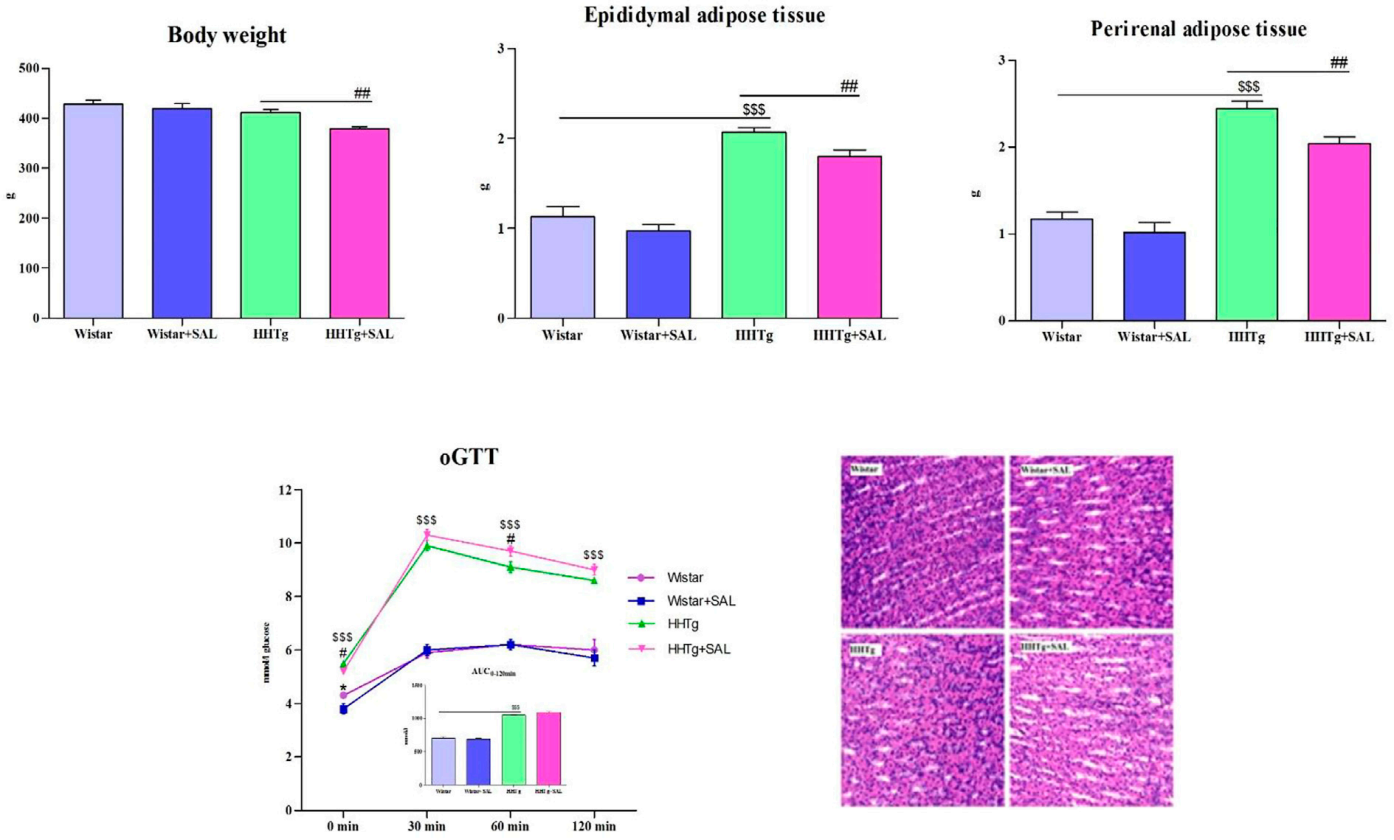
Effect of salsalate treatment on body weight and relative weight of visceral adipose tissues— epididymal and perirenal, and oral glucose tolerance test in Wistar control and hereditary hypertriglyceridemic (HHTg) rats. Values are expressed as mean ± SEM; *n* = 8; Effect of salsalate on histological evaluation of the stomach wall. Representative histological images of intact gastric mucosa of each group showing neither inflammatory changes nor bleeding (hematoxylin and eosin (H&E) staining, 400×). * denotes significance reflecting the effect of Wistar vs. Wistar + SAL, ^#^ denotes significance reflecting the effect of HHTg vs. HHTg + SAL, ^$^ denotes significance reflecting the effect of Wistar vs. HHTg; *p˂0.05; ^#^ *p*˂0.05; ^$$$^ *p*˂0.001.

After salsalate treatment, HHTg rats had lower body weight, while body weight of Wistar rats were not significantly affected (Figure 1). Decreased body weight in HHTg rats was associated with decreased relative weight of visceral adipose tissue when epididymal and perirenal adipose tissue weight was significantly reduced (*p* < 0.01) (Figure 1). Salsalate treatment did not affect food and water intake in both animal groups.

### 3.2 The effects of salsalate on inflammatory parameters

Salsalate-treated Wistar animals exhibited decreased serum inflammatory markers MCP-1 and leptin while salsalate-treated HHTg animals exhibited decreased serum MCP-1 and TNFα (Table 1). On the other hand, serum levels of IL6 and hs CRP in Wistar and HHTg groups were not affected by salsalate administration. No inflammatory changes were observed in histological evaluation in the liver in salsalate-treated animals when compared to controls (Figure 1).

**TABLE 1:** Effect of salsalate treatment on serum parameters in Wistar control and prediabetic HHTg rats.

	Wistar	Wistar + SAL	HHTg	HHTg + SAL	P_S_	P_T_	P_I_
Fasting glucose (mmol/L)	4.3 ± 0.1	3.8 ± 0.2*	5.5 ± 0.1^$$$^	5.2 ± 0.1^#^	0.001	0.01	n.s.
Non-fasting glucose (mmol/L)	7.0 ± 0.2	6.7 ± 0.1	8.3 ± 0.2^$$$^	7.8 ± 0.2^#^	0.001	0.05	n.s.
Insulin (nmol/L)	0.46 ± 0.06	0.16 ± 0.01***	0.24 ± 0.02^$$$^	0.21 ± 0.02	0.05	0.001	0.001
Glucagon (pg/mL)	371 ± 44	184 ± 10***	165 ± 9^$$$^	150 ± 5	0.001	0.001	0.001
HMW adiponectin (µg/mL)	2.87 ± 0.11	2.51 ± 0.15	2.54 ± 0.16	2.29 ± 0.16	n.s.	n.s.	n.s.
TAG (mmol/L)	1.19 ± 0.05	0.88 ± 0.08	5.71 ± 0.18^$$$^	2.24 ± 0.12^###^	0.001	0.001	0.001
Total cholesterol (mmol/L)	1.32 ± 0.07	1.17 ± 0.06	1.52 ± 0.03^$^	1.25 ± 0.04^###^	0.05	0.001	n.s.
HDL-cholesterol (mmol/L)	1.06 ± 0.06	0.94 ± 0.07	0.75 ± 0.02^$$$^	0.81 ± 0.04	0.001	n.s.	n.s.
NEFA (mmol/L)	0.30 ± 0.08	0.28 ± 0.05	0.50 ± 0.02^$^	0.47 ± 0.04	0.001	n.s.	n.s.
Leptin (ng/mL)	7.29 ± 0.42	5.50 ± 0.61*	11.37 ± 0.24^$$$^	11.28 ± 0.49	0.001	n.s.	n.s.
MCP-1 (ng/mL)	283 ± 17	219 ± 25*	244 ± 21	174 ± 10^#^	0.05	0.01	n.s.
TNFα (pg/mL)	2.02 ± 0.13	1.77 ± 0.07	3.75 ± 0.16^$$$^	2.82 ± 0.06^###^	0.001	0.001	0.01
IL-6 (pg/mL)	9.35 ± 1.83	8.41 ± 1.51	12.67 ± 1.05	9.72 ± 0.62	n.s.	n.s.	n.s.
hs CRP (μg/mL)	247 ± 27	282 ± 17	230 ± 17	254 ± 18	n.s.	n.s.	n.s.
ALT (μkat/L)	1.05 ± 0.20	1.11 ± 0.04	1.16 ± 0.13	1.22 ± 0.07	n.s.	n.s.	n.s.
AST (μkat/L)	2.43 ± 0.16	2.72 ± 0.51	2.38 ± 0.21	2.84 ± 0.04	n.s.	n.s.	n.s.

Two-way ANOVA results: P_S_ denotes the significance of Wistar vs. HHTg (strain effects), P_T_ denotes the significance of salsalate (treatment effects); P_I_ denotes the significance of salsalate in both strains (treatment vs. strain interaction). For multiple comparisons Fisher’s LSD *post hoc* test was used; * denotes significance reflecting the effect of Wistar vs. Wistar + SAL, ^#^ denotes significance reflecting the effect of HHTg vs. HHTg + SAL, ^$^ denotes significance reflecting the effect of Wistar vs. HHTg, n.s. denotes not significant; *p˂0.05, ***p˂0.001; ^#^ *p*˂0.05, ^###^ *p* < 0.001; ^$^ *p*˂0.05, ^$$$^ *p*˂0.001. Data are mean ± SEM; *n* = 8 for each group. HHTg, hereditary hypertriglyceridemic rats; TAG, triacylglycerols; MCP-1, monocyte chemoattractant protein; TNFα–tumor necrosis factor α; IL-6, interleukin-6; hs CRP, high-sensitivity C-reactive protein.

### 3.3 The effects of salsalate on oxidative and dicarbonyl stress

Salsalate administration in HHTg rats *versus* untreated controls was associated with reduced oxidative stress in the liver as suggested by increased activity of antioxidant enzymes SOD and GPx as well as decreased levels of oxidized form of glutathione and parameters of lipoperoxidation (Table 2). In addition, salsalate-treated HHTg rats showed reduced dicarbonyl stress when hepatic levels of methylglyoxal were reduced (Figure 4).

**TABLE 2:** Effect of salsalate treatment on hepatic oxidative stress parameters in Wistar control and prediabetic HHTg rats.

	Wistar	Wistar + SAL	HHTg	HHTg + SAL	P_S_	P_T_	P_I_
TBARS, nmol/mg_prot_	1.32 ± 0.13	1.22 ± 0.12	1.64 ± 0.10^$^	1.20 ± 0.06^##^	n.s.	0.05	n.s.
Conjugated dienes, nmol/mg_prot_	32.0 ± 2.4	28.6 ± 2.2	41.6 ± 2.4^$$^	28.8 ± 1.2^###^	0.05	0.001	0.05
GSH, μmol/mg_prot_	68.61 ± 4.33	71.31 ± 4.94	62.89 ± 2.56	73.06 ± 2.96^#^	n.s.	0.05	n.s.
GSSG, μmol/mg_prot_	1.11 ± 0.10	1.27 ± 0.10	1.74 ± 0.16^$$^	1.22 ± 0.10^##^	0.05	n.s.	0.01
GSH/GSSG	62.86 ± 3.11	56.97 ± 2.65	37.49 ± 3.10^$$$^	63.20 ± 6.12^###^	0.05	0.05	0.01
SOD, U/mg_prot_	0.16 ± 0.01	0.17 ± 0.01	0.11 ± 0.01^$$^	0.14 ± 0.01^#^	0.01	0.05	n.s.
GPx, μmol NADPH/min/mg_prot_	276 ± 28	259 ± 20	222 ± 10	301 ± 27^#^	n.s.	n.s.	0.05
GR, nmol NADPH/min/mg_prot_	166 ± 13	210 ± 29	133 ± 12	136 ± 13	0.01	n.s.	n.s.

Two-way ANOVA, results; P_S_, denotes the significance of Wistar vs. HHTg (strain effects), P_T_, denotes the significance of salsalate (treatment effects); P_I_, denotes the significance of salsalate in both strains (treatment vs. strain interaction). For multiple comparisons Fisher’s LSD, *post hoc* test was used; * denotes significance reflecting the effect of Wistar vs. Wistar + SAL, ^#^ denotes significance reflecting the effect of HHTg vs. HHTg + SAL, ^$^ denotes significance reflecting the effect of Wistar vs. HHTg, n.s. denotes not significant; ^#^ *p*˂0.05, ^##^ *p*˂0.01, ^###^ *p* < 0.001; ^$^ *p*˂0.05, ^$$^ *p*˂0.01, ^$$$^ *p*˂0.001. Data are mean ± SEM; *n* = 8 for each group. HHTg, hereditary hypertriglyceridemic rats; GSH, glutathione; GSSG, oxidized form of glutathione; SOD, superoxide dismutase; GPx, glutathioneperoxidase; GR, glutathione reductase; TBARS, thiobarbituric acid-reactive substance.

### 3.4 The effects of salsalate on glucose tolerance and insulin sensitivity

**FIGURE 2:**
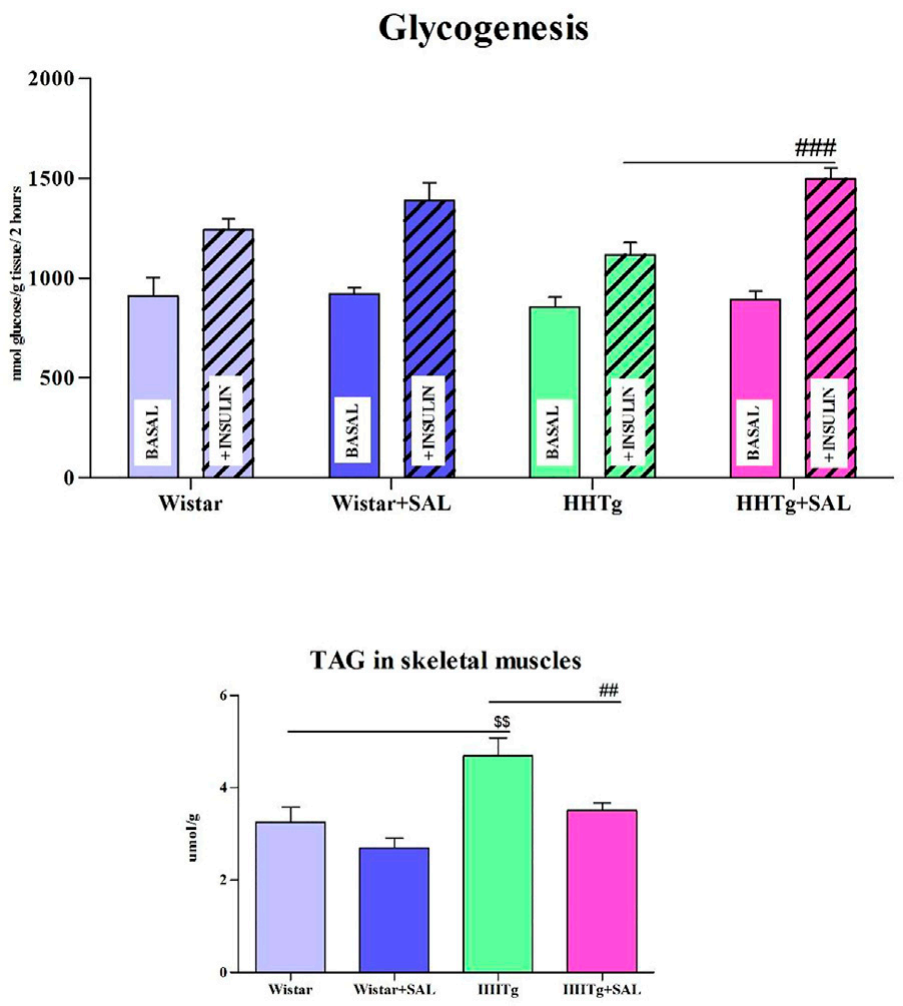
Effect of salsalate treatment on parameters of insulin sensitivity in skeletal muscles. Basal and insulin-stimulated ^14^C-U glucose incorporation into glycogen in muscle (glycogenesis) and skeletal muscle triglycerides concentration in Wistar control and hereditary hypertriglyceridemic (HHTg). Values are expressed as mean ± SEM; *n* = 8; ^#^ denotes significance reflecting the effect of HHTg vs. HHTg + SAL, ^$^ denotes significance reflecting the effect of Wistar vs. HHTg; ^##^ *p*˂0.01, ^###^ *p*˂0.001; ^$$^ *p*˂0.01.

**FIGURE 3:**
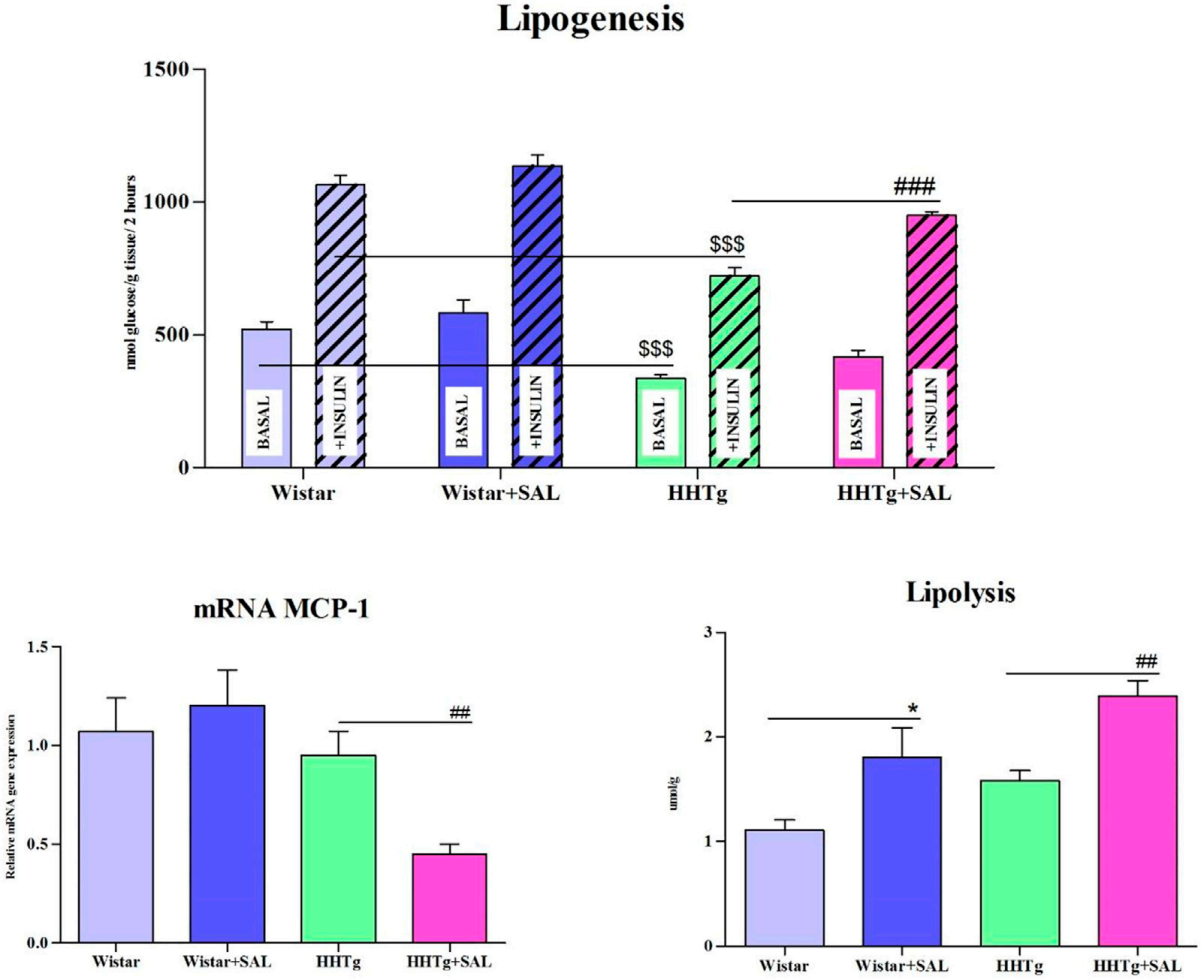
Effect of salsalate treatment on metabolic activity of epididymal adipose tissue. Basal and insulin-stimulated ^14^C-U glucose incorporation into lipids in epididymal adipose tissue (lipogenesis), lipolysis and relative mRNA expression of MCP1 in Wistar control and hereditary hypertriglyceridemic (HHTg) rats. Values are expressed as mean ± SEM; *n* = 8; * denotes significance reflecting the effect of Wistar vs. Wistar + SAL, ^#^ denotes significance reflecting the effect of HHTg vs. HHTg + SAL, ^$^ denotes significance reflecting the effect of Wistar vs. HHTg; *p < 0.05; ^##^ *p*˂0.01, ^###^ *p*˂0.001; ^$$$^ *p*˂0.001.

Fasting as well as non-fasting glucose was decreased in HHTg-treated group and only fasting glucose was decreased in Wistar-treated group when compared to their respective controls (Table 1). On the other hand, salsalate administration did not significantly change AUC for glucose tolerance in both experimental groups (Figure 1). In salsalate-treated Wistar group, the levels of insulin and glucagon were markedly reduced (*p* < 0.001) while no significant effects of salsalate was observed in HHTg rats (Table 1). Epididymal adipose tissue of salsalate-treated HHTg rats was more sensitive to insulin action when insulin-stimulated lipogenesis was significantly increased (*p* < 0.001) *versus* untreated controls (Figure 3). Salsalate treatment was associated with increased lipolytic activity of epididymal adipose tissue in both Wistar and HHTg rats when compared to their respective controls (Figure 3). Skeletal muscles of HHTg rats treated with salsalate contained less triglycerides and showed increased sensitivity to insulin action when insulin-stimulated glycogenesis was significantly higher (*p* < 0.001) compared to untreated controls (Figure 2). There were no significant differences in insulin sensitivity parameters in the Wistar group after salsalate administration.

### 3.5 The effect of salsalate on serum and hepatic lipids

**FIGURE 4:**
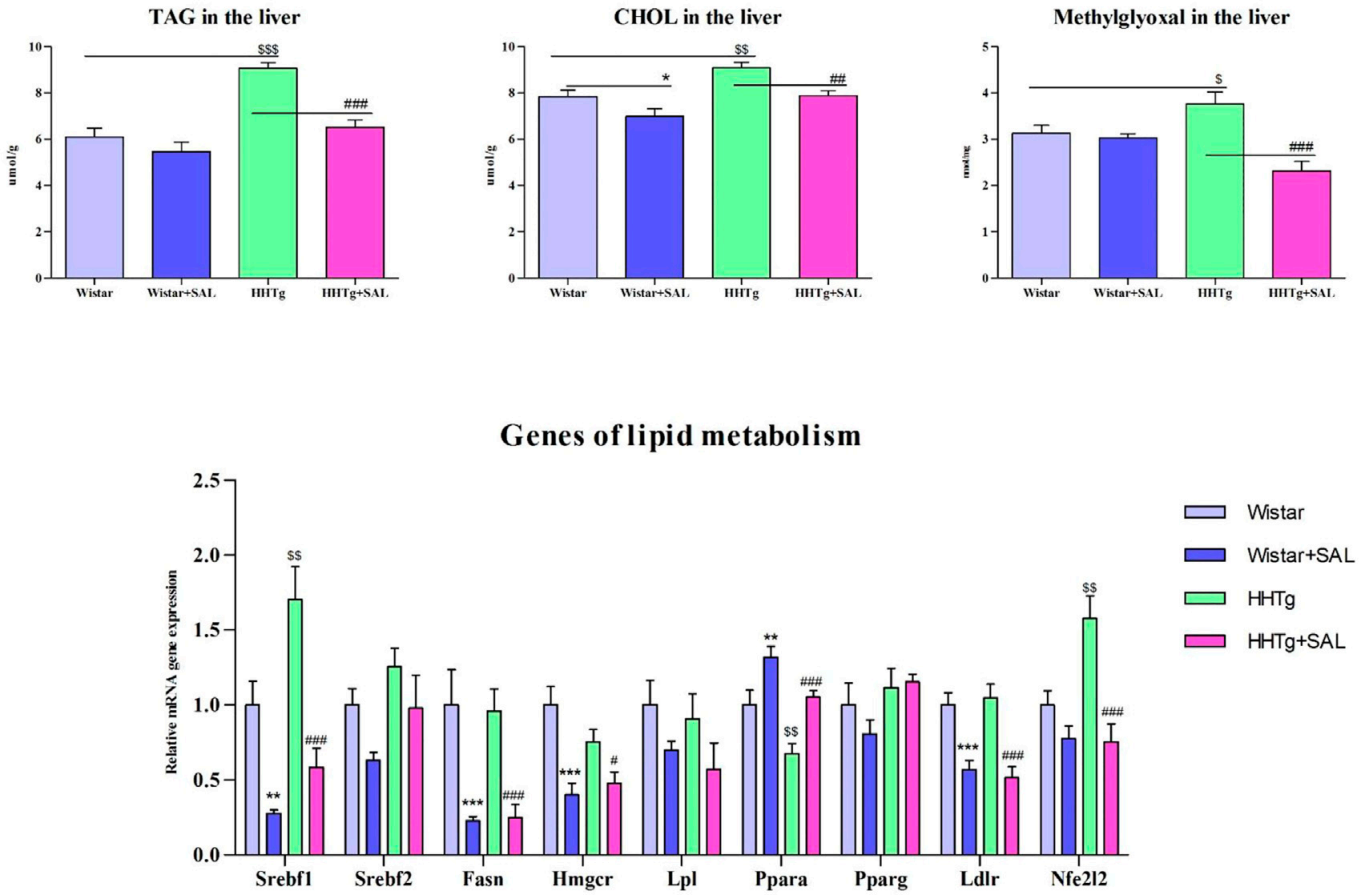
Effect of salsalate treatment on hepatic lipids and methylglyoxal accumulation and relative mRNA expression of enzymes and transcription factors involved of lipid metabolism in Wistar control and hereditary hypertriglyceridemic (HHTg) rats. Values are expressed as mean ± SEM; *n* = 8; * denotes significance reflecting the effect of Wistar vs. Wistar + SAL, ^#^ denotes significance reflecting the effect of HHTg vs. HHTg + SAL, ^$^ denotes significance reflecting the effect of Wistar vs. HHTg; *p < 0.05, **p < 0.01, ***p < 0.001; ^#^ *p*˂0.05, ^##^ *p*˂0.01, ^###^ *p*˂0.001; ^$^ *p*˂0.05, ^$$^ *p*˂0.01, ^$$$^ *p*˂0.001.

After salsalate administration, serum levels of triacylglyceroles and total cholesterol were significantly decreased (*p* < 0.001) in HHTg rats *versus* their untreated controls, but HDL cholesterol and NEFA were not affected (Table 1). Salsalate administration significantly reduced hepatic triglyceride (*p* < 0.001) and cholesterol (*p* < 0.01) concentrations in HHTg rats *versus* untreated controls (Figure 4).

### 3.6 The effects of salsalate on gene expression

**FIGURE 5:**
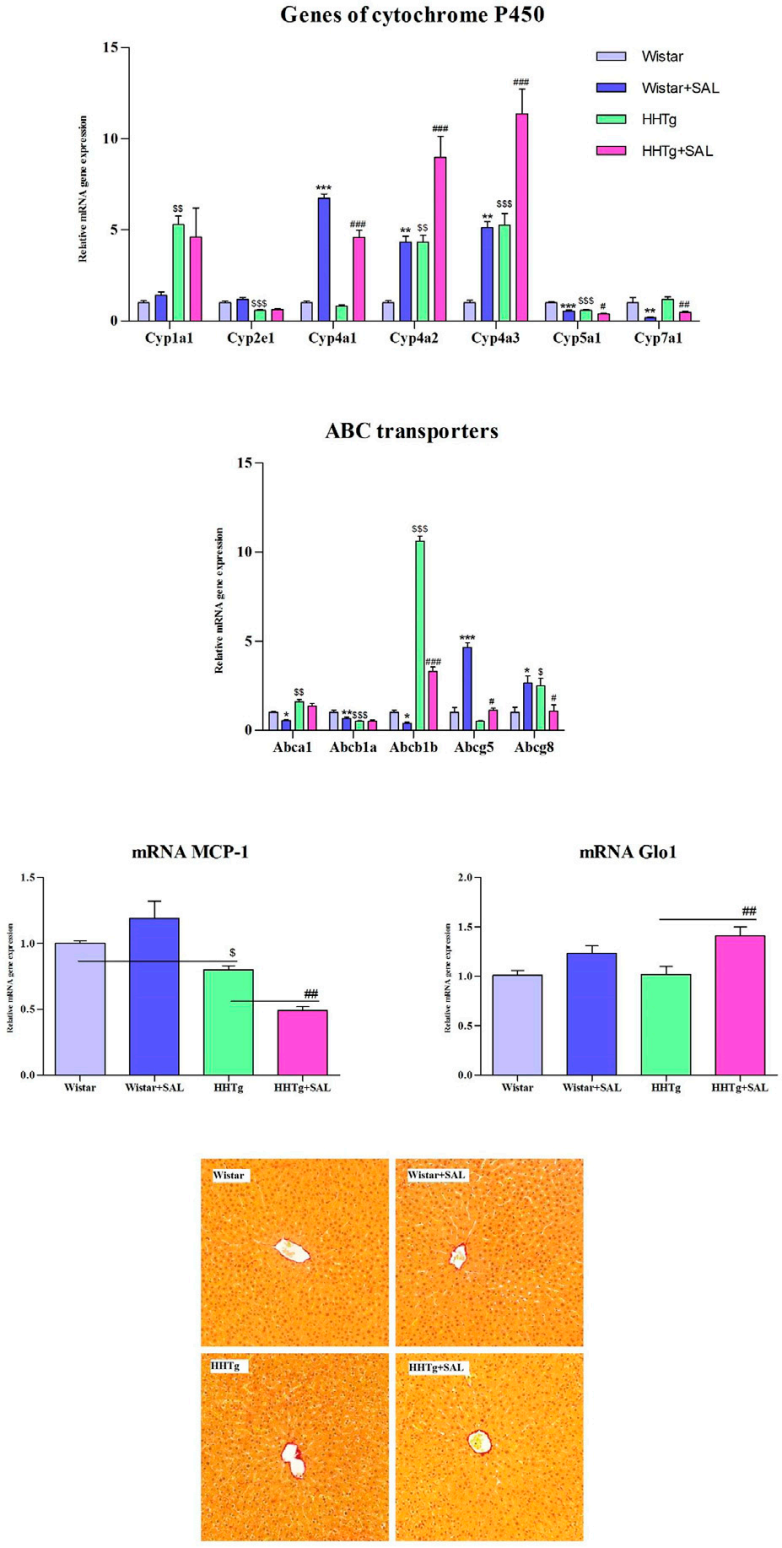
Effect of salsalate treatment on hepatic relative mRNA expression of cytochrome P450 family proteins, ABC transporters, inflammation marker MCP-1 and dicarbonyl stress enzyme Glo-1 in Wistar control and hereditary hypertriglyceridemic (HHTg) rats. Values are expressed as mean ± SEM; *n* = 8; Effect of salsalate on histological evaluation in the liver. Histological images of liver parenchyma (picrosirius red (PSR) staining, 200x). We observed no apparent abnormity in any experimental group. * denotes significance reflecting the effect of Wistar vs. Wistar + SAL, ^#^ denotes significance reflecting the effect of HHTg vs. HHTg + SAL, ^$^ denotes significance reflecting the effect of Wistar vs. HHTg; *p < 0.05, **p < 0.01, ***p < 0.001; ^#^ *p*˂0.05, ^##^ *p*˂0.01, ^###^ *p*˂0.001; ^$^ *p*˂0.05, ^$$^ *p*˂0.01, ^$$$^ *p*˂0.001.

To search for responsible molecular mechanisms of salsalate protective effects against inflammation and metabolic disturbances we determined mRNA abundance of selected genes in tissues. Anti-inflammatory effects of salsalate in HHTg rats compared to untreated controls were associated with reduced expression of *Ccl2* gene (coding for the MCP-1 cytokine) in adipose tissue (*p* < 0.01) and in the liver (*p* < 0.01) (Figures 3, 5). Reduced dicarbonyl stress in the liver after treatment of HHTg rats with salsalate was associated with increased hepatic expression of *Glo1* gene (*p* < 0.01) that codes for an enzyme involved in methylglyoxal degradation.

Amelioration of dyslipidemia in salsalate-treated HHTg rats was associated with reduced hepatic expression of *Fasn* (*p* < 0.001) and *Srebp1* (*p* < 0.001) genes coding for lipogenic enzyme and lipogenic transcription factor, respectively (Figure 4). Reduced hepatic cholesterol content in HHTg rats after salsalate treatment was associated with decreased relative expression of *Hmgcr* gene (*p* < 0.05), coding for an enzyme involved in cholesterol synthesis, and *Ldlr* gene (*p* < 0.001), involved in cholesterol transport into the cells (Figure 4). Salsalate treatment also affected expression of genes coding for hepatic membrane cholesterol transporters—relative mRNA expression of *Abcg5* was significantly elevated (*p* < 0.001 for Wistar group; *p* < 0.05 for HHTg group) while expression of *Abcb1b* gene (*p* < 0.05 for Wistar group; *p* < 0.001 for HHTg group) was markedly decreased in both treated groups compared to untreated controls (Figure 5). In Wistar salsalate-treated rats, mRNA expression of *Abcb1a* and *Abca1* genes was significantly decreased (*p* < 0.01; *p* < 0.05, respectively.). Salsalate treatment increased mRNA expression of *Abcg8* in Wistar group while decreased in HHTg group of rats (Figure 5). Increased gene expression of *Ppara* gene coding for transcription factor in salsalate-treated animals can contribute to increased hepatic lipid oxidation (Figure 4). On the other hand, no differences in mRNA expression of *Pparγ*, *Srebf2* and *Lpl* (Figure 4). Compared to untreated group, salsalted-treated HHTg rats also exhibited decreased mRNA expression of *Nfe212* (Figure 4).

In the liver, salsalate treatment affected expression of some genes coding for cytochrome P450 proteins involved in lipid metabolism. As shown in Figure 5, relative gene expression of *Cyp7a1* and *Cyp5a1* genes was significantly decreased in both salsalate-treated Wistar (*p* < 0.01; *p* < 0.001, respectively.) and HHTg (*p* < 0.01; *p* < 0.05, respectively.) groups of rats whereas markedly elevated relative gene expression of *Cyp4a1*, *Cyp4a2*, *Cyp4a3* was observed in salsalate-treated animals (*p* < 0.001; *p* < 0.01, respectively.) (Figure 5). On the other hand, no differences in relative gene expression of *Cyp1a1* and *Cyp2e1* were observed in both Wistar and HHTg rats (Figure 5). According to histological evaluations of liver parenchyma we observed no apparent abnormity in any experimental group (Figure 5).

## 4 Discussion

In the present study, we investigated metabolic effects of salsalate and examined responsible mechanisms of these effects in HHTg rats, a prediabetic model with hypertriglyceridemia and insulin resistance. Insulin resistance—a typical sign of prediabetic state was accompanied by changes in lipid metabolism, increased oxidative stress and the presence of low-grade inflammation. HHTg rats exhibit all disorders associated with prediabetes but without the complications of high-fat-diet-induced obesity. Our results demonstrate that salsalate treatment improved glycaemia, insulin sensitivity and exhibited hypolipidemic properties.

The anti-inflammatory effects of salsalate are believed to be attributable to inhibition of pro-inflammatory mediators such as prostaglandins (Morris, et al., 1985). Salsalate treatment is associated with inhibition of the COX activity, a key enzyme in biosynthesis of pro-inflammatory prostanoids, but also inhibition of neutrophil activation and strongly inhibits NFκB inflammatory pathway (Rumore and Kim, 2010). Transcription factor NFκB is the master switch of the inflammatory pathway and stimulating its activity in adipose tissue and the liver results in insulin resistance (Goldfine, et al., 2008). In the present study, salsalate treatment exhibited anti-inflammatory effect in circulation, as well as in visceral adipose tissue and liver. Decreased MCP-1 expression in adipose tissue and liver can reduce macrophage infiltration and can affect other pro-inflammatory gene expression. These effects can ameliorate insulin sensitivity and NAFLD progression.

Beneficial effects of salsalate on lipid metabolism in the liver may be related to reduce oxidative and dicarbonyl stress in HHTg rats. Salsalate treatment ameliorated oxidative stress by stimulating antioxidant enzyme activity that can inactivate free radicals in the liver directly. In addition, increased activity of SOD and GPx may play a role in decreasing hepatic lipid peroxidation by participating in the removal of lipoperoxidation products. Increased GPx activity after salsalate treatment was also linked to increased levels of glutathione, a sensitive marker of hepatic oxidative damage. Markedly increased GSH/GSSG ratio following salsalate administration can mitigate oxidative as well dicarbonyl stress. Reduced methylglyoxal accumulation can occur due to increased gene expression of glutathione-dependent *Glo-1*, enzyme involved in methylglyoxal degradation. Dicarbonyl stress may be involved in the pathogenesis of hepatic steatosis and methylglyoxal can contribute *via* several mechanisms to the development of hepatic steatosis. Methylglyoxal decreases glutathione levels, induces lipoperoxidation, may impair insulin signaling and activates RAGE receptors (Huttl, et al., 2020). In addition, some inflammatory pathways, in particular those related to NFκB, are activated by methylglyoxal (Neves, et al., 2019). According to our previous results (Malinska, et al., 2021), hepatic methylglyoxal concentrations well correlated with hepatic triglycerides concentrations. Thus, mitigation of dicarbonyl stress through decreased methylglyoxal and increased *Glo1* expression, can protect against NAFLD development and/or progression.

Our results showed that salsalate administration improved glucose tolerance and insulin sensitivity in peripheral tissues. Glucose-lowering effect of salsalate was previously observed in diabetic (Goldfine, et al., 2010) as well in prediabetic individuals (Faghihimani, et al., 2012), however the exact mechanism was not clarified yet. It was suggested that salsalate suppresses hepatic glucose production, positively affects insulin and glucagon and protects β-cells (Penesova, et al., 2015; Han, et al., 2019). Smith et al. suggested that the mechanism by which salsalate improves glucose homeostasis and NAFLD is *via* salicylate-driven mitochondrial uncoupling (Smith, et al., 2016). Antidiabetic effect of salsalate may be also related to reducing intestinal inflammation and improved gut dysbiosis that was observed in ZDF rats (Zhang, et al., 2020). Although several studies have found increased adiponectin levels after salsalate administration (Wang, et al., 2014; Salastekar, et al., 2017), which could contribute to improvement of insulin sensitivity, in the present study salsalate did not affect adiponectin levels. It has been also suggested that the IKKβ pathway is a target for insulin sensitizing effects of salsalate (Yuan, et al., 2001).

Severe hepatic lipid accumulation in HHTg rats was ameliorated following salsalate treatment when both hepatic triglyceride and cholesterol concentrations were markedly reduced. Lower ectopic fat accumulation could contribute to increased insulin sensitivity and reduced lipid peroxidation. Compared to the anti-inflammatory and antidiabetic effects, the hypolipidemic effects are more pronounced in the prediabetic HHTg model. Higher efficacy of salsalate in the presence of serious dyslipidemia and hepatic lipid accumulation is promising for a potential use of salsalate in prediabetic patients with NAFLD.

Our analysis revealed differential hepatic expression of selected genes that may be responsible for hypolipidemic effects of salsalate. These are genes coding for enzymes or transcription factors which are involved in lipid synthesis (*Fas*, *Hmgcr*, *Srebpf1*), oxidation (*Ppara*) as well as transport into the cells (*Ldl* receptors, *Abc* transporters). Accordingly, reduction of hepatic lipid accumulation can be a product of decreasing *de novo* synthesis as well as increasing lipid oxidation. Decreased gene expression of transcription factor *Srebp1* can reduce hepatic lipid accumulation *via* suppression of lipogenesis, in turn contributing to improved insulin sensitivity.

Salsalate may modulate fatty acid oxidation, lipid synthesis, and lipoprotein metabolism. Recently, salsalate has been reported to directly activate AMP-activated protein kinase, which plays an important role in the regulation of inflammation and hepatic lipid metabolism (Hawley, et al., 2012). Salsalate was found to potently suppress hepatic *de novo* lipogenesis *in vitro* and *in vivo* (Smith et al., 2016). A recent report indicates that salicylates reduced hepatic NFκB activity and hepatic VLDL triglycerides production in high-fat fed mice (Baker, et al., 2011). Other study with diet-induced NASH in mice (Liang et al., 2015) revealed an important role for PPARα-mediated pathways with regards to the beneficial effects of salsalate on lipid metabolism. In our previous study in SHR-CRP rats, a model of inflammation and metabolic disturbances, we also observed increased hepatic expression of *Ppara* gene after salsalate treatment that was associated with reduced ectopic fat accumulation in the liver (Trnovská et al., 2017).

Abcg5/8 transporters not only play an important role in sterol absorption and excretion but also provide an important pathway for cholesterol elimination (Poruba, et al., 2019). Increased gene expression of *Abcg8* transporter indicated higher cholesterol secretion from hepatocytes into bile. Beyond a role in cellular cholesterol homeostasis, ABC transporters can be involved in the secretion and bioavailability of key molecules controlling glucose and lipid metabolism, such as lipoprotein lipase or insulin (Hardy, et al., 2017). Thus, modulation of ABC transporters can associate with metabolic disorders including T2DM and insulin resistance. In the present study, in a prediabetic HHTg model with severe hypertriglyceridemia we observed markedly increased expression of *Abcb1b*, *Abca1*, and *Abcg8* genes and decreased expression of *Abcb1*a and *Abcg5* genes.

In the present study, salsalate treatment was associated with alterations in hepatic expression of genes coding for cytochrome P450 family proteins, especially those that can be associated with lipid metabolism and fatty liver development. Hepatic lipid accumulation in HHTg rats was associated with markedly increased hepatic gene expression of *Cyp1a1*, *Cyp4a2* and *Cyp4a3* and decreased *Cyp2e1* and *Cyp7a1* that can be involved in hepatic lipid dysmetabolism and fatty liver development as was reviewed by Jamwal and Barlock 2020.

According to recent reports, CYP4A isoforms are markedly elevated in the liver tissues of NAFLD patients (Ryu, et al., 2019), as well as in diabetic and obese mice with hepatic steatosis (Zhang, et al., 2017). CYP4A cytochromes metabolize EETs (epoxyeicosatrienoic acids) to HEETs (hydroxy-epoxyeico-satrienoic acids) and act as PPAR-α agonists. Increased CYP4A after salsalate treatment can contribute to increased fatty acid oxidation in the liver through increased transcription factor PPARα (Zhang and Klaassen, 2013). The effect of salsalate on CYP4a proteins in our study is in agreement with results in model with essential hypertension and chronic inflammation, the SHR-CRP rats. In this study, SHR rats with transgenic expression of human CRP exerted increased gene expression of *Cyp4a1* in the liver following salsalate administration that was also associated with increased *Ppara* gene expression (Trnovska, et al., 2017).

In our study, salsalate treatment was associated with reduced gene expression of *Cyp5a1*–CYP450 isoform that metabolizes the cyclooxygenase product prostaglandin H2 into thromboxane A2, a potent inducer of vasoconstriction and platelet aggregation. (Ershov, et al., 2021). Thus, it is possible that salsalate can improve vascular complications also through decreased CYP5a1.

The hepatic CYP7a1 protein is a rate-limiting enzyme responsible for cholesterol conversion to 7α-hydroxycholesterol, which is secreted into bile acids and eliminated (Chiang and Ferrell, 2020). Since hepatic expression of *Cyp7a1* gene was reduced in HHTg rats after salsalate treatment, we assume that decreased hepatic cholesterol concentrations after salsalate administration are not due to increased elimination, rather to decreased hepatic synthesis. Taken together, changes in hepatic gene expressions of CYP family proteins following salsalate treatment may be involved in reducing lipid accumulation in the liver. Possible study limitation as well as the strength may be related to the used strain of rats because the found results can be applied to specific group of patients in human medicine. However, this is consistent with the requirements of personalized medicine.

## 5 Conclusion

Salsalate treatment in HHTg rats markedly reduced inflammation, oxidative and dicarbonyl stress, ameliorated hepatic lipid metabolism and ectopic lipid deposition. These effects were associated with increased insulin sensitivity in peripheral tissues and protect against the progression and/or development of NAFLD and its symptoms. The mechanism of beneficial effect of salsalate on metabolic disorders associated with prediabetic conditions may consist of the changes in cytochrome P450 proteins and the alleviation in oxidative and dicarbonyl stress in the liver. In contrast to the anti-inflammatory effect, hypolipidemic and insulin sensitizing effects of salsalate were more pronounced in the prediabetic HHTg model which suggests that salsalate can have potential beneficial use in prediabetic patients with NAFLD symptoms.

## Data Availability

The datasets presented in this study can be found in online repositories. The names of the repository/repositories and accession number(s) can be found in the article/supplementary material.

## Abbreviations

COX, cyclooxygenase; Fas, fatty acid synthase; Glo-1, glyoxalase-1; GPx, glutathione peroxidase; GSH, reduced form of glutathione; GSSG, oxidized form of glutathione; GR, glutathione reductase; HHTg, hereditary hypertriglyceridemic; Hmgcr, 3-hydroxy-3-methylglutaryl-coenzym A reductase; IL-6, interleukin 6; Ldlr, LDL receptors; MCP-1, monocyte chemoattractant protein-1; NAFLD, non-alcoholic fatty liver disease; NFκB, nuclear factor κB; Pparα, peroxisome proliferator-activated receptor α; RAGE, receptor for advanced glycation end-products; SOD, superoxide dismutase; Srebpf, sterol regulatory element-binding protein; TAG, triacylglyceroles; TNFα, tumor necrosis factor α.
